# Trehalose inhibits ferroptosis via NRF2/HO-1 pathway and promotes functional recovery in mice with spinal cord injury

**DOI:** 10.18632/aging.204009

**Published:** 2022-04-10

**Authors:** Fangyi Gong, Ting Ge, Jing Liu, Jin Xiao, Xiaochuan Wu, Hehui Wang, Yingchun Zhu, Dongdong Xia, Baiwen Hu

**Affiliations:** 1 Department of Orthopedics, Ningbo First Hospital, Ningbo, China; 2 Department of Emergency Medicine, Ningbo First Hospital, Ningbo, China

**Keywords:** spinal cord injury, trehalose, ferroptosis, NRF2/HO-1, neuroinflammation

## Abstract

Spinal cord injury (SCI) is the main cause of severe damage to the central nervous system and leads to irreversible tissue loss and neurological dysfunction. Ferroptosis is a cell death pattern, newly discovered in recent years. Ferroptosis is an oxidizing cell death induced by small molecules, and is an iron-dependent process caused by the imbalance between the generation and degradation of lipid reactive oxygen species (ROS) in cells. As an antioxidant, trehalose can effectively prevent lipid peroxidation. Studies have reported that trehalose can improve the prognosis of SCI. However, it is unclear whether these benefits are related to ferroptosis. In this study, we demonstrated for the first time that trehalose reduces the degeneration and iron accumulation of neurons by inhibiting the production of ROS and ferroptosis caused by lipid peroxides after SCI, thus promoting the survival of neurons and improving the recovery of motor function. More specifically, we found that trehalose inhibited the expansion of cavities in the nerve tissue of mice with SCI, inhibited neuron loss, and improved functional recovery. In terms of mechanism, our results indicate that the neuroprotective effect of trehalose is due to the activation of the NRF2/HO-1 pathway, which in turn inhibits ferroptosis and ferroptosis-related inflammation. Our findings provide important insights into the previously unknown role of trehalose in SCI, as well as new evidence supporting the hypothesis that suppression of ferroptosis plays a key neuroprotective role in SCI.

## INTRODUCTION

Spinal cord injury (SCI) is a common central nervous system injury, manifested by direct injury to the spinal cord and/or nerve roots in the spinal canal, resulting in varying degrees of motor, sensory and autonomic nerve functions loss below the injury plane [1]. The consequences of SCI are devastating and lifelong, inflicting huge pain and heavy burden to patients and their families [2]. In the past 30 years, its global prevalence has increased from 236 to 1,298 cases per million population. The global incidence of SCI is estimated to be between 250,000 and 500,000 per year [3]. At present, the bottleneck in the repair of SCI is effectively dealing with the massive cell death caused by secondary injury. Therefore, the effective control of secondary injury after SC1 and the promotion of the survival of the remaining neurons may be the key to protect the nerve function of the damaged spinal cord. At present, the mechanism of secondary injury is still unclear, but ROS generation and lipid peroxidation after SCI are important causes of secondary injury [4].

Cell death is the result of various physical and chemical factors, and includes programmed cell death and non-programmed cell death. Current studies have found that apoptosis, necroptosis, autophagy, etc., are all programmed cell death [5]. Ferroptosis is a newly defined non-apoptotic programmed cell death mode. This concept was first proposed by Stockwell et al. in 2012, and further studies have successively reported ferroptosis-related features [6]. Ferroptosis is different from other programmed cell death modes in terms of morphology, biochemistry, gene expression and regulation. Mitochondrial shrinkage and reduction or disappearance of mitochondrial cristae are typical cell morphological features of ferroptosis [7]. Increased intracellular iron content and lipid ROS levels are other features. High levels of ROS initiate lipid peroxidation, leading to the breakdown of lipid membranes and cell death [8]. Ferroptosis can be triggered by small molecule activators or other conditional factors. Its main feature is to inhibit glutathione synthesis or the activity of glutathione peroxidase 4 (GPX4). After GPX4 activity is reduced, it cannot inhibit the production of ROS in the cell, leading to lipid peroxidation and ROS aggregation, thus leading to cell death [6].

In the acute phase of SCI, the destruction of the blood-spinal cord barrier and blood vessels cause substantial bleeding, which may even last for several days [9]. Bleeding leads to an increase in the concentration of iron at the site of injury; therefore, the Fenton reaction mediated by iron can generate a large amount of ROS, which further initiates the lipid peroxidation reaction [10]. Studies have shown that GSH, one of the main antioxidant components in cells, significantly decreases the content of lesions after SCI [11]. Yao et al. confirmed the existence of ferroptosis in the early stage of SCI, and found that the use of the iron-chelating agent, deferoxamine, can inhibit the occurrence of ferroptosis and promote motor functions recovery in rats [12]. Zhang et al. used the ferroptosis specific inhibitor SRS 16–86 to treat rats with SCI and found that it can reverse the changes in mitochondria, reduce lipid peroxidation products, and enhance endogenous antioxidant capacity, thereby inhibiting the occurrence of ferroptosis and promoting recovery of nerve function in rats [13]. In addition, studies have shown that carnosic acid can inhibit the ferroptosis of PC12 cells induced by Erastin by activating the NRF2 pathway, suggesting that carnosic acid can play a neuroprotective effect in the inhibition of ferroptosis occurrence [14]. The latest research findings indicate that both zinc and proanthocyanidin can inhibit ferroptosis, thereby promoting functional recovery after SCI [15, 16]. Therefore, in-depth exploration of the mechanism of ferroptosis in SCI and the search for new intervention targets provide certain guidance for combined treatment of SCI.

Trehalose is a non-reducing disaccharide composed of two D-glucose units connected to α-1,1, and is widely distributed in many non-mammalian organisms, including bacteria, fungi, plants and invertebrates [17]. Under various stress conditions, such as oxidative damage, dehydration and temperature changes, trehalose exerts a cytoprotective effect [18]. Studies have shown that trehalose provides effective neuroprotection in animal models with chronic central nervous system diseases (such as Huntington’s disease, Alzheimer’s disease and amyotrophic lateral sclerosis) [19, 20]. Previous studies reported that trehalose can improve the prognosis of SCI [21, 22]. However, it is unclear whether these benefits are related to ferroptosis. Several studies have shown that trehalose, being an antioxidant, effectively prevents lipid peroxidation [23–25]. These scientific findings suggest that trehalose may improve functional recovery after SCI by affecting ferroptosis. Therefore, in this study, we analyzed the protective effect of trehalose on SCI, as well as the role of NRF2 pathway and ferroptosis in this hypothetical protective effect.

## MATERIALS AND METHODS

### SCI model and trehalose treatment

The mice were first anaesthetized by intraperitoneal injection of 2% pentobarbital, before fully exposing the spinous process and lamina of the T10 segment. After fully exposing the spinal cord, a striker rod with a chosen weight of 10 g and a height of 2.5 mm was released to fall freely for spinal cord percussion modelling. Hemorrhage and swelling of the spinal cord in the striking segment can be seen immediately after the blow. The criterion for evaluating the success of the SCI model is: after modeling, twitching of the lower limbs of the mouse is first seen, which in turn leads to loss of muscle strength and paralysis. The wound was rinsed with saline and sutured layer by layer. After modeling, the mice were made to urinate artificially everyday, once a day, until their autonomous urination function was restored. Trehalose (Sigma) was prepared as a 2% w/v solution in a standard suspension vehicle (SSV; 0.9% NaCl, 0.5% Na-carboxy-methylcellulose, 0.5% benzyl alcohol and 0.4% Tween 80). The animals were given 0.1 ml/10 g body weight by gavage daily for three days.

### Function scoring test

#### BMS (basso mouse scale) score

On the 1st, 3rd, 7th, 14th, 21st and 28th days after injury, all mice were observed by two independent researchers who were blind to the treatment group. The BMS score ranges from 0 (no ankle movement) to 9 (full functional recovery), and the movement was scored based on hind limb joint movement, trunk position and stability, step coordination, paw position, toe gap, and tail position.

#### Footprint test

On the 28th day after injury, gait and motor coordination were evaluated. The front paws and hind paws were painted with different colors of dye. Then, the mouse was placed on a piece of blotting paper surrounded by a cage to encourage it to walk in a straight line. Footprint patterns were digitized, and representative images were used to assess coordination.

#### Incline plate test

The 1st, 3rd, 7th, 14th, 21st and 28th days after injury were taken as the time points to observe the recovery of the nerve function of the mice: The test surface had a special inclined plate made of rubber with shallow grooves, and the ability of the mouse to maintain the posture, their grasping ability and the maximum angle at which they stayed on the inclined plate for at least 5 seconds were measured as its function value.

#### Rotating rod test

The animal was placed on a stationary rotating rod for 180 seconds, and then the rotation speed was accelerated from 5 times/min to 40 times/min within 5 minutes. Within 390 seconds of the subsequent test, the time at which the mouse fell from the rotating rod for the first time was recorded. Each test was performed 3 times, with an interval of 5 minutes, and the average value was taken.

#### Swimming test

The swimming test was used to evaluate the recovery of motor function of the mice after SCI. In short, the mice were trained to swim from one end of a glass tank filled with water to the other. The Louisville Swimming Scale was used to assess forelimb dependence, hindlimb movement and alternation, trunk instability, and body angle. Each mouse needed to be tested twice, and the final score was based on the average score of the two trials.

### Hematoxylin-eosin (HE) staining

The section was placed in Harris’ solution to stain the nucleus. The staining time was about 5–10 minutes. After the nucleus was stained, the section was rinsed with running water, 1% hydrochloric acid-alcohol was used for a few seconds for differentiation, and then the section was rinsed again with running water before using 0.6% ammonia water to restore the blue colour, and once this was achieved, tap water was used to rinse it. Then the stained section of the nucleus was immersed in eosin and allowed to stain for about 3 minutes. Hematoxylin-eosin staining was followed by gradient dehydration, which used gradient alcohol for dehydration (95% ethanol, 11–100% alcohol, 100% alcohol-xylene-xylene, each for 5 minutes), transparent, and then the dehydrated slices were taken out, and neutral gum was used to mount the slices. HE slice images were observed, collected and analysed.

### Fluor Jade B (FJB) staining

Before staining, the sections were placed at room temperature for at least 30 minutes. Then, the slices were immersed in an alkaline ethanol solution containing 1% sodium hydroxide and 80% ethanol for 5 minutes. They were then soaked in 70% ethanol for 2 minutes, rinsed with distilled water for 2 minutes, and then incubated in 0.06% potassium permanganate solution for 10 minutes. After rinsing with water for 1–2 minutes, the slide glass was transferred to 0.0004% Fluor Jade B solution (Millipore), dissolved in 0.1% acetic acid solvent for 10 minutes. Then the slides were rinsed three times with distilled water, ensuring that each rinsing lasted 1 minute. The slides were then air-dried for at least 5 minutes. FJB positive cells were counted under a microscope, and NIH Image J software was used for analysis.

### Perls staining

As mentioned before [26], the accumulation of iron in cells was detected by Perls staining. In short, the sections were immersed in distilled water for 3 minutes, then placed in Perl solution (5% potassium ferrocyanide/5% hydrochloric acid) (Sigma) for 30 minutes, and then washed in phosphate buffered saline (PBS). The endogenous peroxidase activity was blocked by a 0.3% methanol solution of hydrogen peroxide for 15 minutes, and then washed three times in PBS. 3,3-diaminobenzidine and hematoxylin were used for counter-staining. A randomly selected field of view was observed under a microscope and photographed, and iron-positive cells were counted.

### Dihydroethidium (DHE) staining

The ROS probe dihydroethidium (DHE) fluorescence staining method was used to assess ROS levels. The sections were washed with PBS solution and replaced with freshly prepared 10 μM DHE (GeneCopoeia) containing PBS, protected from light, at 37°C for 40 minutes. The cells were observed and photographed under a fluorescence microscope (Zeiss, Heidenheim, Germany).

### ELISA

As per the manufacturer’s instructions, the ELISA method was used to detect the content of TNF-α, IL-1β and IL-6 in the cerebrospinal fluid, spinal cord tissue and cell supernatant. The absorbance was measured at 450 nm using a microplate reader (BioTek, Friedrichshall, Germany). The cerebrospinal fluid was directly extracted through the needle through the foramen magnum, and the cerebrospinal fluid was transferred into a 1.5 mL EP tube, centrifuged at 12,000 g/min to remove blood cells, and stored in liquid nitrogen.

### Fe^2+^, MDA, GSH measurement

As per the manufacturer’s protocol, iron content kit, lipid peroxide product MDA kit and GSH kit (Abcam) were used to detect iron content, lipid peroxide MDA and GSH expression in tissues or cells, respectively.

### Extraction of neuronal cells

New-born C57/BL6 mice were taken, decapitated with ophthalmic scissors and their brains were exposed. The inner half-moon-shaped hippocampal tissue of the mouse was slowly exposed and separated using a microscope, and then the tissue was placed in D-Hanks solution containing double antibodies. The tissue was cut and made into a paste with microscissors. The hippocampal tissue fragments of the mouse were added to 2 ml of 0.125% membrane protease, and incubated at 37°C for 20 minutes, while being shaken once every 5 minutes. Then 2 ml of Dulbecco’s Modified Eagle Medium (DMEM) culture (Thermo Fisher Scientific, MA, USA) containing 10% FBS was added to the tissue to stop the digestion, 140 microliters of DNAase (Sigma-Aldrich, MO, USA) was also added, and the cells were blown with a pipette until they were completely decomposed, leaving no visible tissue masses behind. The tissue suspension was filtered with a 200-mesh cell screen, and the cell suspension was centrifuged at 1000 rpm for 5 minutes. The supernatant was removed, the cells resuspended in Neurobasal medium for neuronal cells, pipetted until they were completely dispersed, and inoculated in the culture plate. They were cultivated in an incubator containing 5% CO_2_ at 37°C. 24 hours after cell inoculation, Neurobasal medium + B27 (Thermo Fisher Scientific) was replaced in full for culture, and half of the medium was changed every 2–3 days.

### Western blot

As per the manufacturer’s protocol, a whole protein extraction kit was used to extract proteins from cells and tissues. After protein quantification by BCA method, 1xSDS was added and boiled in water for 5 minutes. The same amount of protein was transferred to PVDF membrane, then to sodium dodecyl sulfate polyacrylamide gel (SDS-PAGE), and lastly to 5% milk blocking for 1 hour, with anti-Acsl4 (Abcam, 1:5000), anti-Gpx 4 (Abcam, 1:5000), anti-IL-6 (Proteintech, 1:1000), anti-IL-1β (Proteintech, 1:1000), anti-TNF-α (Abcam, 1:1000), anti-HO-1 (Proteintech, 1:2000), anti-NRF2 (Affinity, 1:1000), and anti-GAPDH (Affinity, 1:10000). A secondary antibody was added, and it was incubated for 1 hour at 4°C overnight. The PVDF membrane was developed by ECL method (Pierce Biotechnology, MA, USA) and exposed to light, and the results were analyzed by Image J software.

### Immunofluorescence

Spinal cord tissue or cultured neuronal cells were fixed with 4% paraformaldehyde for 15 minutes, then infiltrated with 0.25% Triton X-100 for another 15 minutes, and blocked with 10% normal goat serum for 1 hour. They were incubated with anti-NRF2 (Affinity, 1:200), anti-NeuN (Abcam, 1:400), anti-GFAP (Abcam, 1:5000), anti-CD11b (Abcam, 1:200) at 4°C overnight, then Alexa Fluor 488- and Alexa Faur 594-conjugated goat secondary antibodies (Jackson ImmunoResearch) were incubated for 1 hour at room temperature. After washing with PBS three times, the nuclei were stained with DAPI (Beyotime, China), and fluorescence images were obtained using a fluorescence microscope (Zeiss, Heidenheim, Germany).

### RNA isolation and qPCR

The total RNA of the cells and spinal cord were extracted using Trizol reagent (Takara, Dalian, China) and then converted into cDNA using the reverse transcription kit HiScript II Q RT SuperMix for qPCR (R122-01, Vazyme, China). Thereafter, according to the manufacturer’s instructions, in the 7500 real-time PCR system (Applied Biosystems, Inc., USA), the AceQ qPCR SYBR green master mix (Q111-02, Vazyme, China) was used to perform the qPCR reaction. The mRNA level of the target gene was normalized to the expression of glyceraldehyde-3-phosphate dehydrogenase (GAPDH). The qPCR results were quantified by 2^−ΔΔCt^ methods. Primer Sequence was available on request.

### Cell viability assessment

The neuronal cells were seeded into a 96-well plate, with 3 replicate wells in each group. After corresponding treatment, the liquid was discarded in the wells, 190 uL culture medium and 10 uL CCK8 working solution (Dojindo) were added to each well, incubation continued for 4 hours, shaken on a horizontal shaker for 15 minutes, and each well was measured with a microplate reader (BioTek, Friedrichshall, Germany) at 450 nm wavelength OD value. The relative survival rate of each group of cells was calculated.

### PI staining

After the neuron cells were processed accordingly, PI staining solution (Beyotime, China) was added, according to the manufacturer’s protocol, observed under a fluorescence microscope, and photographs were taken for recording purposes.

### ROS

The cell ROS analysis kit (ab186027, Abcam) was used to quantify the production of ROS in cells using a fluorescent microplate reader, according to the manufacturer’s protocol. The excitation and emission wavelengths were 520 and 605 nm, respectively. Analysis was performed by flow cytometry (FACS Canto II, BD Biosciences). FlowJo software (Version 7.6.1, Treestar, Ashland, OR, USA) was used for data analysis.

### Statistical analysis

GraphPad Prism 8.0 software was used for data processing, statistical analysis, and statistical chart production. The measurement data is expressed in the form of mean ± standard deviation (x ± s). The statistical results of Tukey’s post-hoc test was used, and Student’s *T*-test was used for comparison between the two groups. *P* < 0.05 was regarded as statistically significant.

## RESULTS

### Trehalose promotes the recovery of motor function after SCI in mice

**Figure 1:**
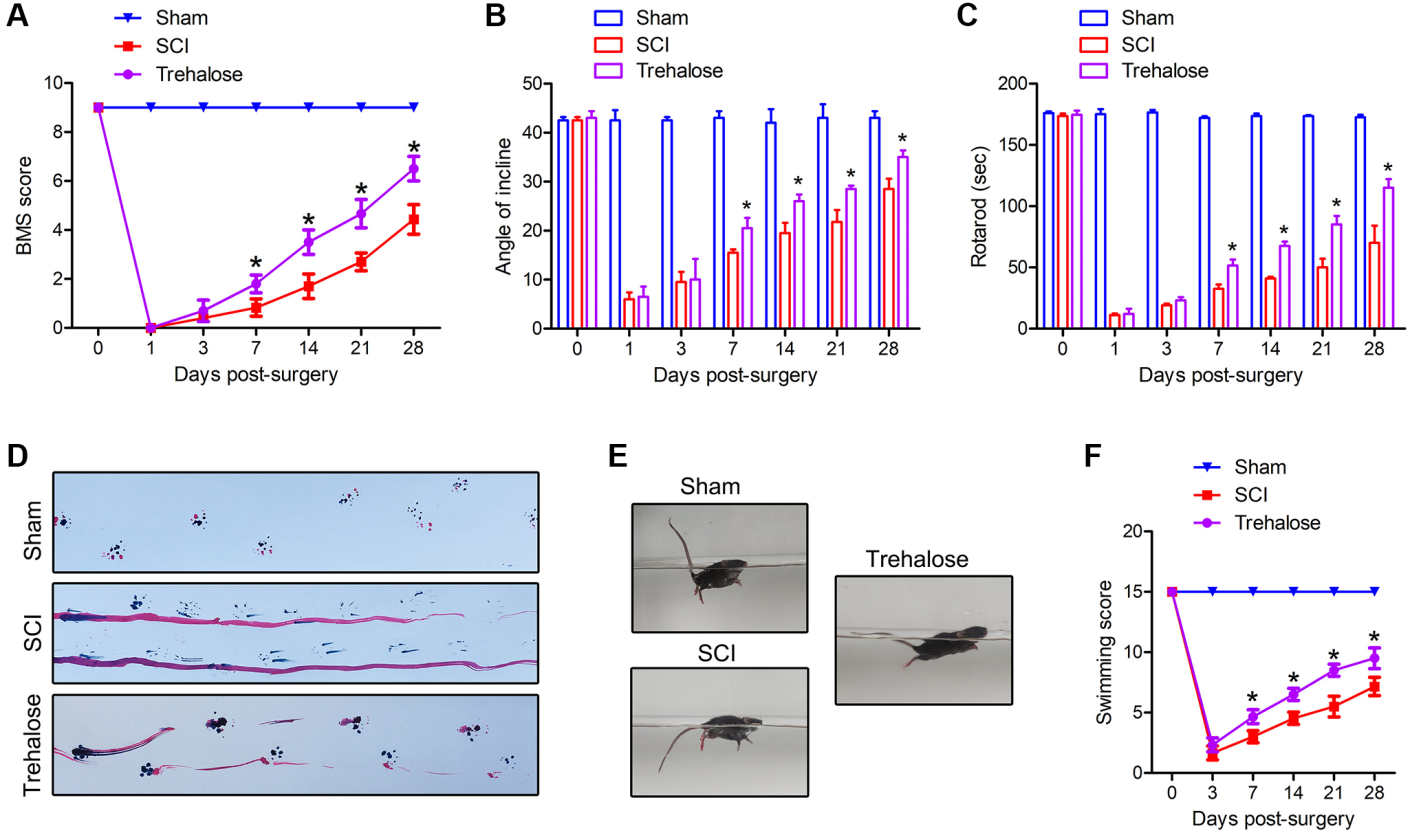
**Trehalose promotes the recovery of motor function after SCI in mice.** (**A**) BMS scores on day 1, 3, 7, 14, 21 and 28 after SCI; (**B**) Inclined plate test on day 1, 3, 7, 14, 21 and 28 after SCI; (**C**) Rotating rod test on day 1, 3, 7, 14, 21, and 28 after SCI; (**D**) Footprint test on the 28th day after SCI; (**E**, **F**) Swimming test. ^*^p < 0.05 compared with the SCI group.

In order to study the effect of trehalose on the functional recovery of mice with SCI, BMS scores, inclined plate test and rotating rod test were used to evaluate the motor function of mice on the 1st, 3rd, 7th, 14th, 21st and 28th days after surgery. The results showed that compared with the SCI group, the BMS score of the trehalose group showed a significant improvement from the first week after the operation (Figure 1A). The inclined plate test and the rotating rod test also confirmed that the motor score of the trehalose group was significantly improved compared with the SCI group starting 1 week after SCI (Figure 1B, 1C). In addition, we performed a footprint test on mice 28 days after injury. After the injury, the coordination of the front paw, hind paw and gait movement of all animals decreased significantly. Compared with the SCI group, the mice treated with trehalose showed a significant improvement in gait recovery and motor coordination (Figure 1D). The Louisville Swim Scale also showed that mice in the trehalose group began to show less forelimb dependence, faster hindlimb alternation, and a smaller angle between the body and the water surface on the 7th day after injury (Figure 1E, 1F). In short, the above experiments show that after SCI, trehalose treatment can promote the recovery of motor function of injured mice.

### Trehalose reduces the damage area and motor neuron loss of SCI in mice

**Figure 2:**
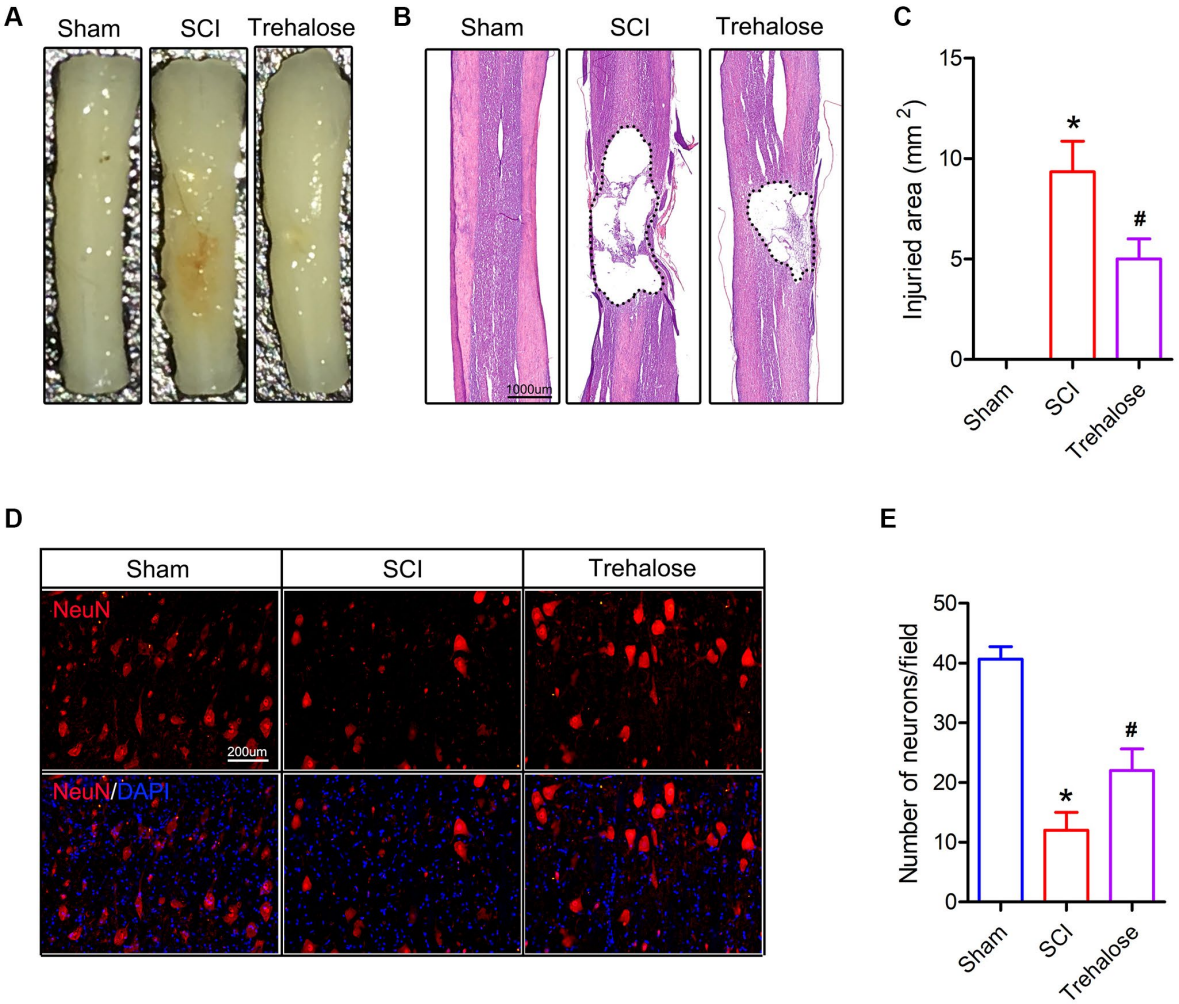
**Trehalose attenuates the damaged area and motor neuron loss of SCI in mice.** (**A**) General picture of the spinal cord on day 28 after SCI; (**B**) HE staining of the spinal cord on day 28 after SCI; (**C**) Quantification of damage area; (**D**) NeuN staining around the spinal cord injury area on the 28th day after SCI; (**E**) Quantitative analysis of neuronal cells. ^*^p < 0.05 compared with the Sham group, ^#^*p* < 0.05 compared with the SCI group.

Subsequently, we used HE staining and NeuN staining for histological analysis of spinal cord tissue. On the 28th day after injury, the volume of the injured area and the number of neurons around it were observed. On the 28th day after injury, the spinal cord diagram showed that the area of SCI in the trehalose group was significantly smaller than that of the SCI group (Figure 2A). Similarly, on the 28th day after SCI, HE staining showed that the size of the damaged area in the algalose treatment group was also significantly smaller than that of the SCI group (Figure 2B, 2C). In addition, in order to study the effect of trehalose on surviving neurons after SCI, we performed NeuN staining on the spinal cord tissue around the area of injury on the 28th day after injury. The results showed that the number of neurons around the spinal cord injury area in the SCI group was significantly reduced, and the trehalose treatment showed more surviving neuronal cells than the SCI group, indicating that trehalose can reduce neuronal damage after SCI (Figure 2D, 2E). These data indicate that trehalose treatment can reduce the area of damaged spinal cord injury in mice, and promote tissue preservation and neuronal cell survival after SCI.

### Trehalose inhibits neuronal degeneration and iron accumulation after SCI

**Figure 3:**
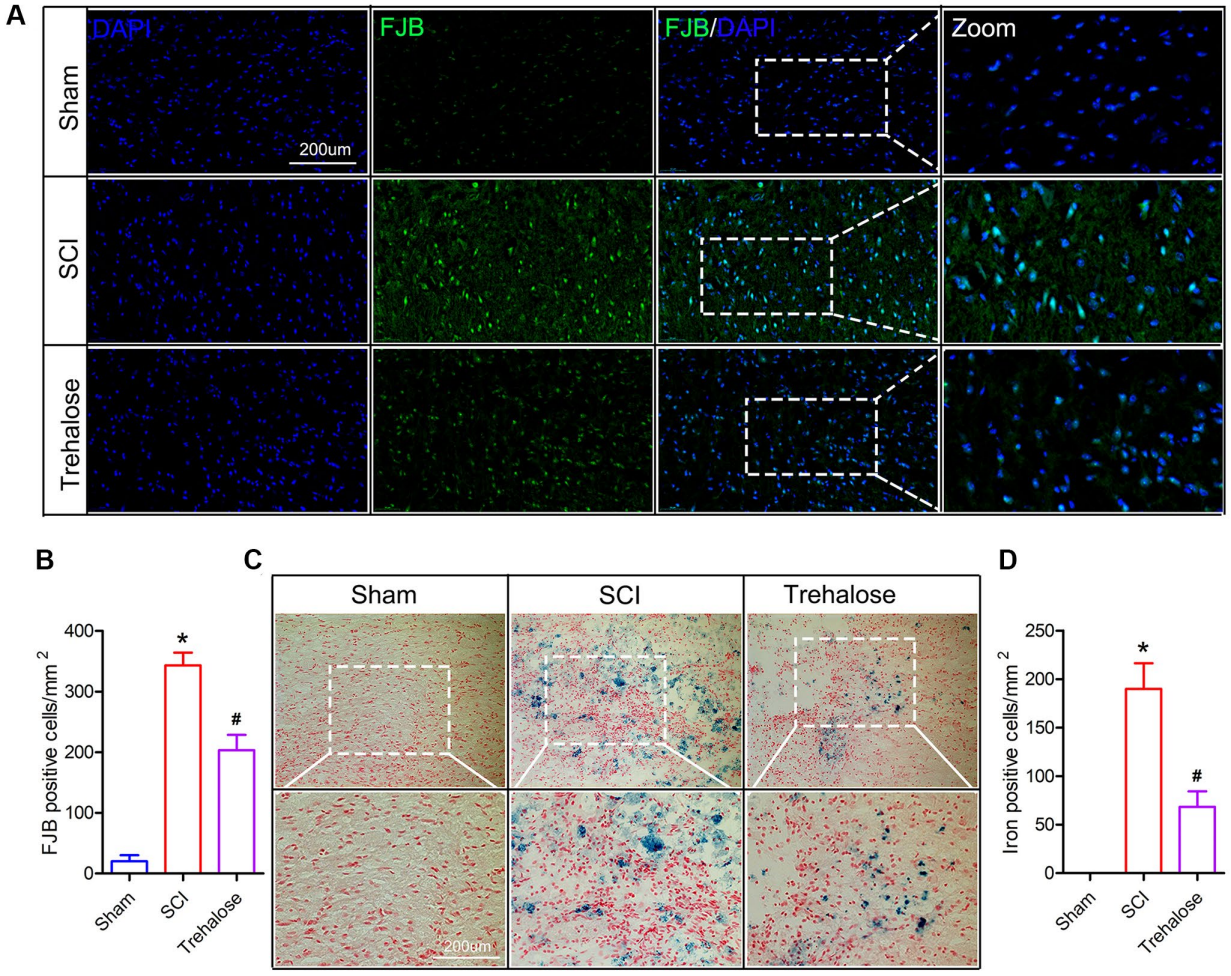
**Trehalose inhibits neuronal degeneration and iron accumulation after SCI.** (**A**) On the third day after SCI, FJB stained area of interest and representative images in spinal cord slices; (**B**) Quantification of FJB positive cells; (**C**) On the third day after SCI, Perls stained region of interest and representative images in spinal cord slices; (**D**) Quantification of iron-positive cells; ^*^p < 0.05 compared with the Sham group, ^#^*p* < 0.05 compared with the SCI group.

FJB staining is a specific staining for degeneration and necrosis of neurons. It is used to compare the degeneration and necrosis of neurons in each group on the 3rd day after SCI. The FJB staining results showed that on the third day after surgery, the FJB positive cells in the spinal cord tissue of the SCI group were significantly increased compared to that in the sham group; and compared to the SCI group, the FJB positive cells in the spinal cord tissue of the trehalose group decreased (Figure 3A, 3B). In addition, we found that on day 3 after SCI, the number of iron-positive cells in the SCI group increased significantly, while compared to the SCI group, the iron-positive cells in the spinal cord tissue of the trehalose group decreased (Figure 3C, 3D).

### Trehalose inhibits the production of ROS and lipid peroxides after SCI

**Figure 4:**
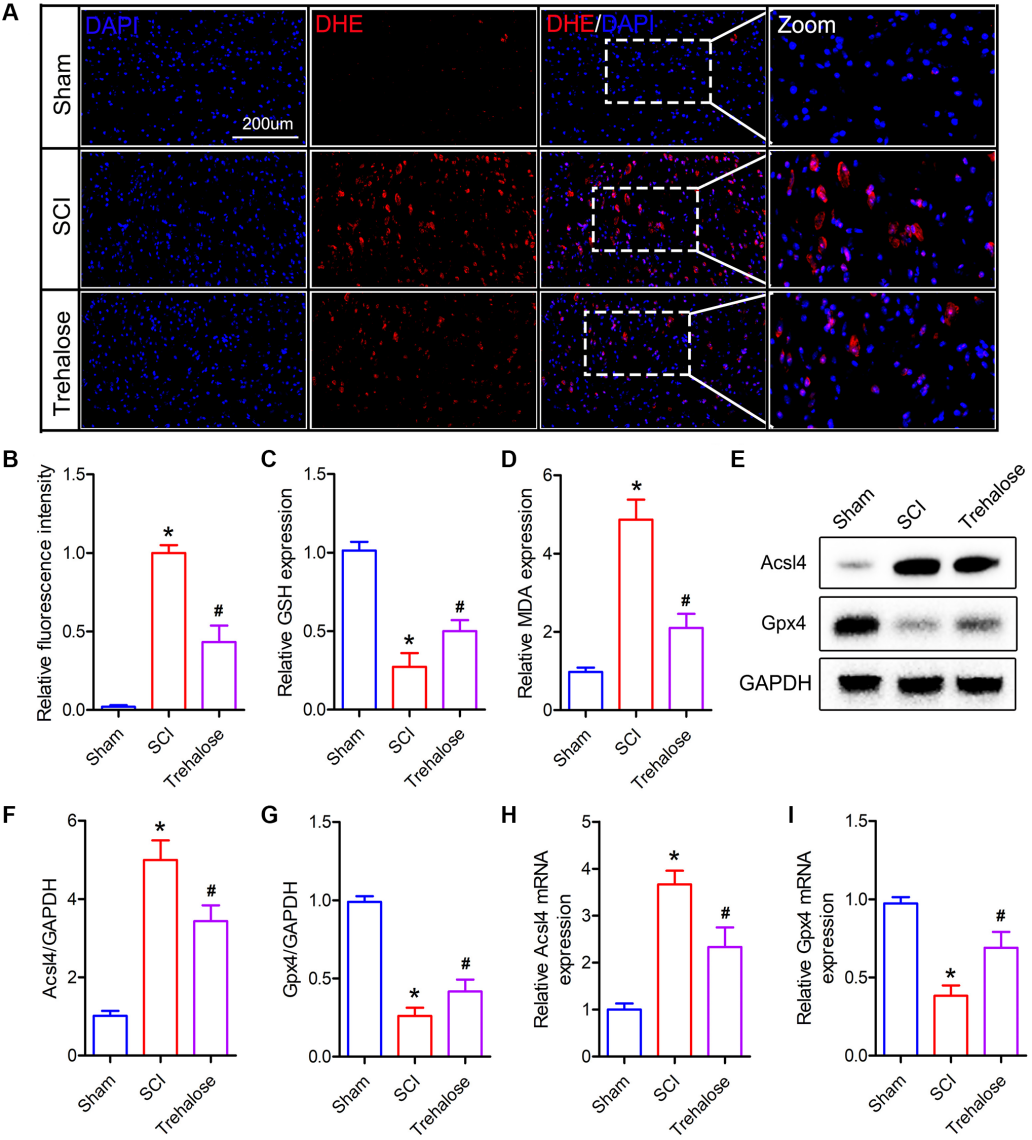
**Trehalose inhibits the production of ROS and lipid peroxides after SCI**. (**A**, **B**) DHE staining of spinal cord tissue on day 3 after SCI and fluorescence quantitative analysis; (**C**) GSH measurement; (**D**) MDA measurement; (**E**–**G**) Western blot to detect the expression of Acsl4 and Gpx4 protein and quantitative protein analysis; (**H**, **I**) PCR to measure the expression of Acsl4 mRNA and Gpx4 mRNA. ^*^p < 0.05 compared with the Sham group, ^#^*p* < 0.05 compared with the SCI group.

Furthermore, we studied the effect of trehalose on ferroptosis-related indicators after SCI. Firstly, we used DHE staining to detect the production of ROS after SCI. The results showed that ROS increased after SCI, while the fluorescence intensity of ROS decreased significantly after treatment with trehalose (Figure 4A, 4B). In addition, the results of lipid peroxide measurement showed that the MDA content in the spinal cord tissue of the SCI group was significantly higher than that in the sham group on the third day after the operation (Figure 4D). After the trehalose intervention, it significantly reduced the level of MDA in the spinal cord of mice with SCI. In addition, the experimental results also showed that the GSH content in the spinal cord tissue of the SCI group was significantly lower than that in the sham group on the third postoperative day, but compared with the SCI group, the GSH content in the spinal cord tissue of the trehalose group was significantly improved (Figure 4C). Then, we used Western blot to measure ferroptosis-related proteins and found that trehalose treatment can inhibit the decrease of Gpx4 protein and the increase of Acsl4 protein (Figure 4E–4G). PCR further confirmed the Western blot results (Figure 4H, 4I). In summary, the above results prove that trehalose can inhibit ROS production and ferroptosis caused by lipid peroxides after SCI and reduce neuronal cell degeneration and iron accumulation.

### Trehalose inhibits neuroinflammation after SCI

**Figure 5:**
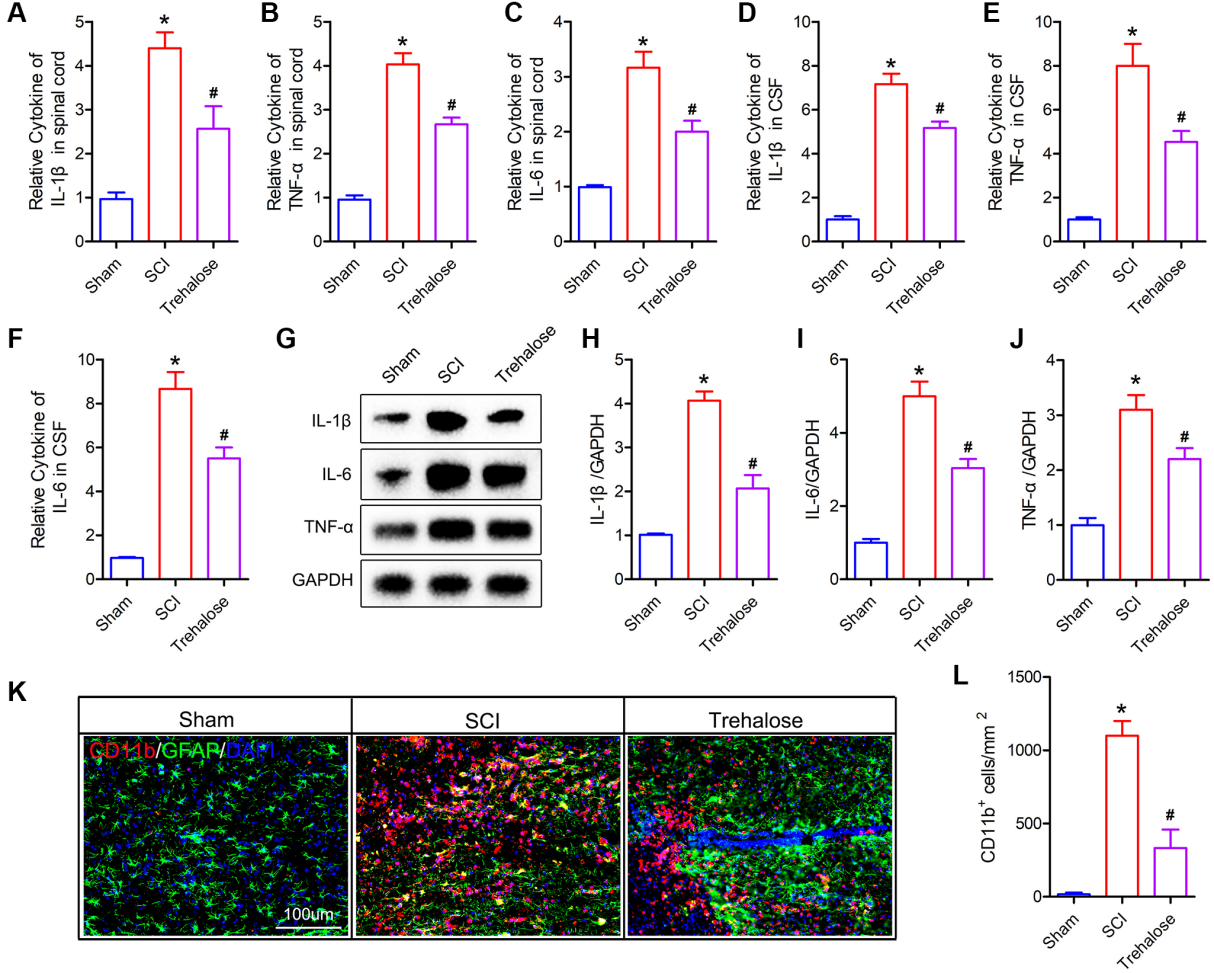
**Trehalose inhibits neuroinflammation after SCI.** (**A**–**C**) ELISA measures the expression of IL-1β, IL-6 and TNF-α in the spinal cord tissue; (**D**–**F**) ELISA measures the expression of IL-1β, IL-6 and TNF-α in CSF; (**G**–**J**) Western blot to detect the expression of IL-1β, IL-6 and TNF-α in spinal cord tissue and quantitative protein analysis; (**K**) CD11b/GFAP immunofluorescence staining; (**L**) Quantitative of CD11b positive cells. ^*^p < 0.05 compared with the Sham group, ^#^*p* < 0.05 compared with the SCI group.

Studies have shown that ferroptosis is closely related to inflammation, and the process of ferroptosis is often accompanied by inflammation, which is called ferroptosis-related inflammation [27]. Therefore, we further studied the effect of trehalose on the inflammatory response after SCI. Firstly, we used the ELISA test to detect pro-inflammatory cytokines in spinal cord tissue and cerebrospinal fluid (CSF). The results showed that 3 days after SCI, IL-1β, IL-6 and TNF-α in both CSF and spinal cord tissue were significantly higher than those in the sham group (Figure 5A–5F). After treatment with trehalose, the levels of IL-1β, IL-6 and TNF-α decreased (Figure 5A–5F). Similarly, Western blot results also confirmed that trehalose treatment can reduce the expression of IL-1β, IL-6 and TNF-α after SCI (Figure 5G–5J). In addition, we used immunofluorescence staining of the microglia marker CD11b to study the effect of trehalose on the activation of microglia. The results showed that compared with the SCI group, the incidence of CD11b positive cells in the trehalose treatment group was significantly reduced (Figure 5K, 5L). In summary, the above results indicate that trehalose can inhibit the neuroinflammatory response after SCI.

### Trehalose inhibits Erastin-induced neuronal cell ferroptosis

**Figure 6:**
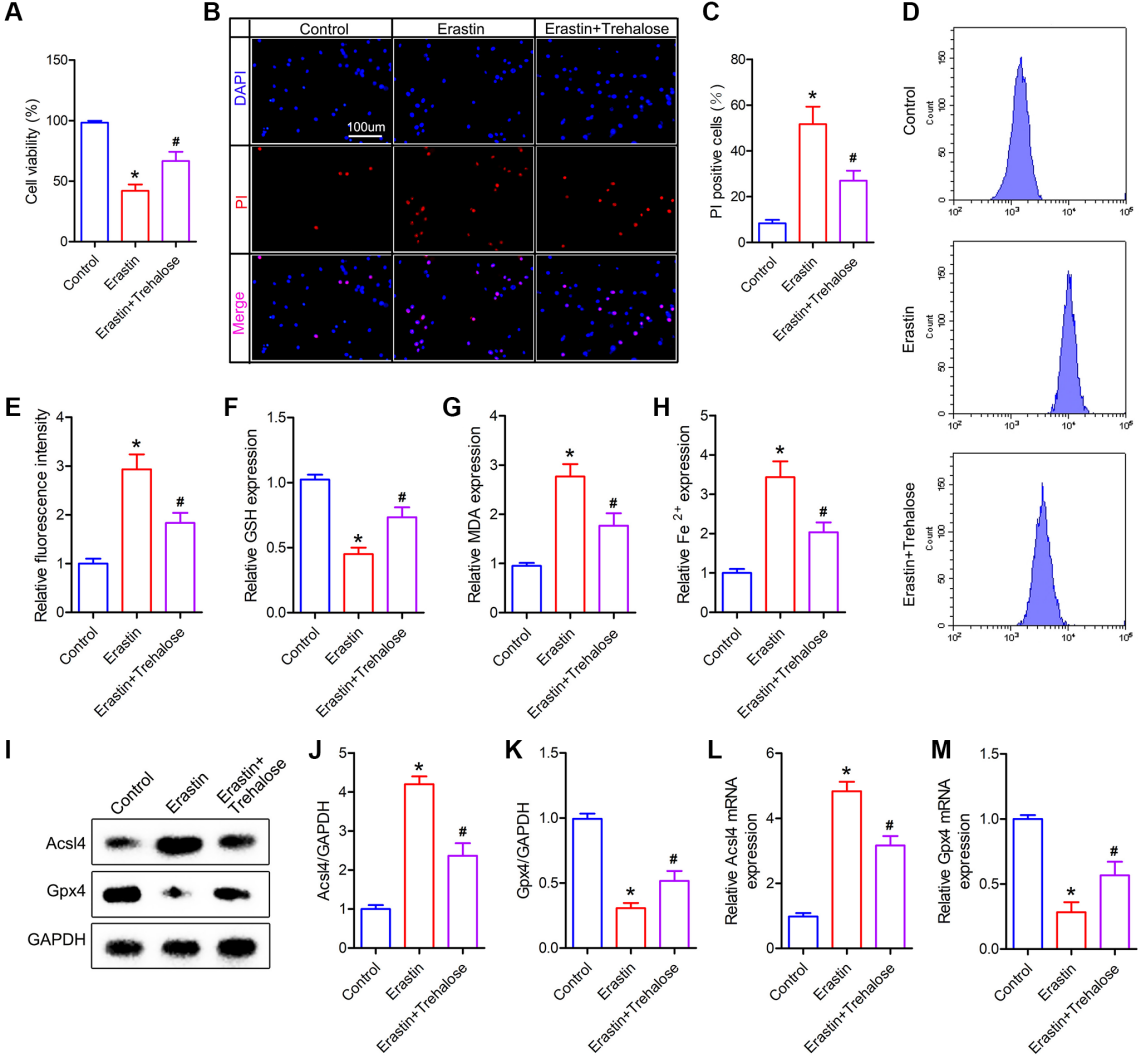
**Trehalose inhibits the ferroptosis of neuronal cells induced by Erastin.** (**A**) CCK8 method is used to assess cell viability; (**B**) PI staining of neuronal cells; (**C**) Quantification of PI positive cells; (**D**, **E**) Flow cytometry to detect ROS level and quantitative analysis; (**F**–**H**) GSH, MDA and Fe^2+^ measurement; (**I**–**K**) Western blot to detect the expression of Acsl4 and Gpx4 protein and quantitative protein analysis; (**L**, **M**) PCR to detect Acsl4 mRNA and Gpx4 mRNA expression. ^*^p < 0.05 compared with the Control group, ^#^*p* < 0.05 compared with the Erastin group.

In order to further verify the neuroprotective effect of trehalose, we pretreated neuronal cells with trehalose (100 μM, 24 h) *in vitro*, and then treated neuronal cells with ferroptosis inducer Erastin (5 μM, 12 h). CCK8 and PI staining were used to detect the viability of neuronal cells. The results of CCK8 showed that the viability of neuronal cells decreased significantly after treatment with Erastin alone, while pretreatment with trehalose could inhibit the decrease in cell viability caused by Erastin (Figure 6A). Similarly, the results of PI staining also showed that trehalose pretreatment inhibited Erastin-induced neuronal cell death (Figure 6B, 6C). The accumulation of large amounts of lipid ROS in cells is a typical feature of ferroptosis, and it is also a key factor in inducing ferroptosis. In order to explore the effect of trehalose on the level of ROS in neuronal cells, we used flow cytometry to detect changes in the expression level of ROS in each group of cells. It was found that trehalose inhibited the increase in ROS levels induced by Erastin (Figure 6D, 6E). We then measured some other relevant indicators of ferroptosis. The results showed that Erastin treatment of neuronal cells significantly increased the level of intracellular MDA and reduced the content of intracellular GSH; pretreatment with trehalose can reverse the effect induced by Erastin (Figure 6F, 6G). In addition, compared with the control group, Erastin can significantly increase the Fe^2+^ content in neuronal cells, but trehalose pretreatment can reduce the increased Fe^2+^ level in the cells (Figure 6H). Lastly, we used Western blot and PCR to detect the expression of ferroptosis-related proteins and found that trehalose pretreatment can inhibit Erastin-induced decrease in Gpx4 expression and increase in Acsl4 expression (Figure 6I–6M). In conclusion, the above results prove that trehalose can inhibit the ferroptosis of neuronal cells induced by Erastin and exert a neuroprotective effect.

### Trehalose inhibits ferroptosis by activating the NRF2/HO-1 signaling pathway

**Figure 7:**
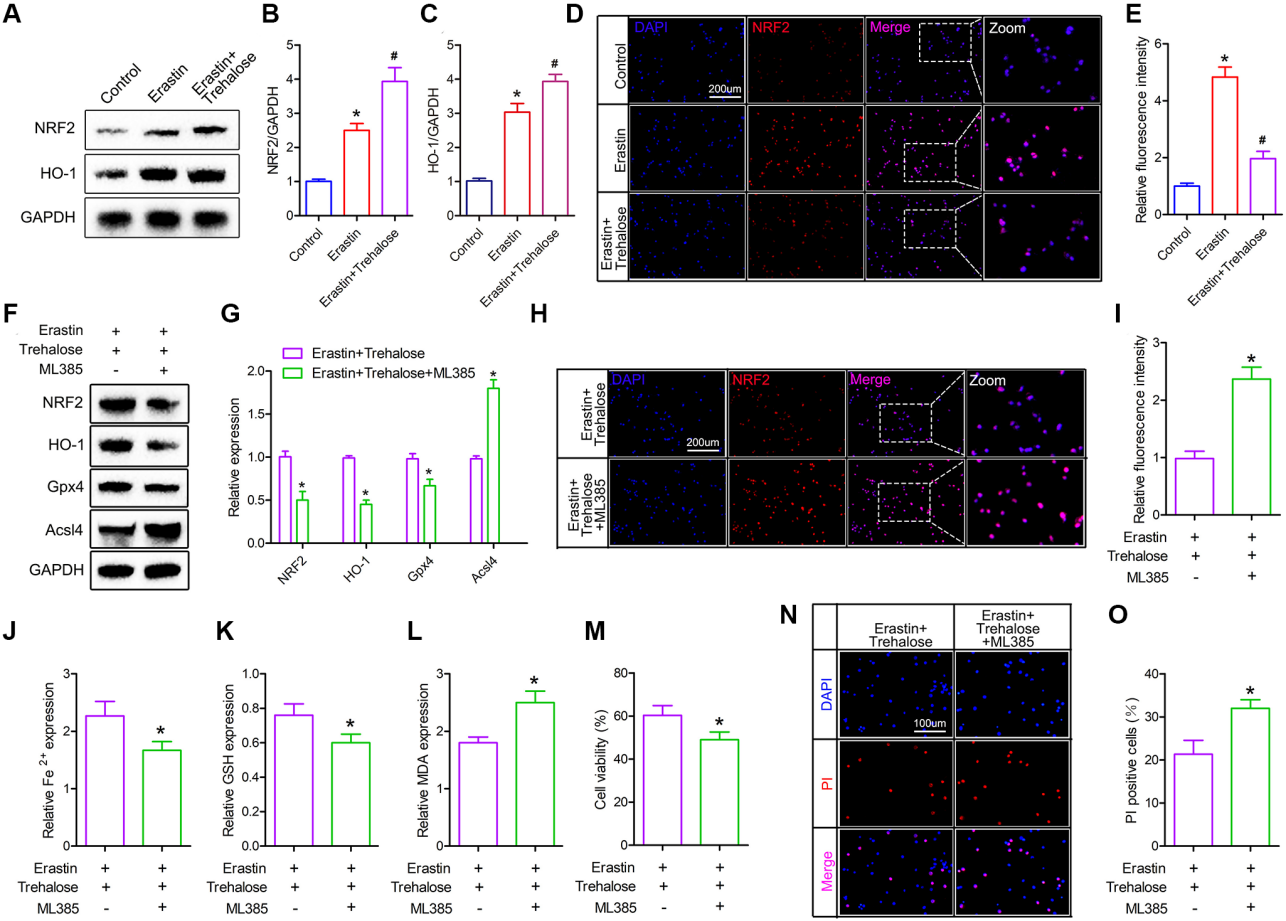
**Trehalose inhibits ferroptosis by activating the NRF2/HO-1 signaling pathway.** (**A**–**C**) Western blot to detect the expression of NRF2 and HO-1 protein and protein quantitative analysis; (**D**, **E**) Immunofluorescence evaluation of NRF2 expression and fluorescence quantification analysis; (**F**, **G**) Western blot to detect the expression of NRF2, HO-1, Acsl4 and Gpx4 protein and quantitative protein analysis; (**H**, **I**) Immunofluorescence to evaluate the expression of NRF2 and quantitative fluorescence analysis; (**J**–**L**) Fe^2+^, MDA and GSH measurement; (**M**) CCK8 method is used to assess cell viability; (**N**) PI staining of neuronal cells; (**O**) Quantification of PI positive cells. ^*^p < 0.05 compared with the Control group or Erastin+Trehalose group, ^#^*p* < 0.05 compared with the Erastin group.

The NRF2 signaling pathway is a direct downstream pathway of ROS, which regulates the transcription of ARE-dependent genes to balance oxidative mediators and maintain cell redox homeostasis. Therefore, we investigated whether the NRF2/HO-1 pathway plays a key role in the inhibitory effect of trehalose on ferroptosis. First, we tested the changes of NRF2 and HO-1 proteins and found that their expression was increased after Erastin treatment, and the high expression of NRF2 and HO-1 proteins was further noticed after trehalose pretreatment (Figure 7A–7C). In addition, immunofluorescence staining of NRF2 also confirmed that trehalose pretreatment promoted the high expression of NRF2 protein (Figure 7D, 7E). To further verify that trehalose inhibits Erastin-induced ferroptosis by activating the NRF2 pathway, we pretreated neuronal cells with trehalose (100 μM, 24 h) and NRF2 pathway inhibitor ML385 (5 μM, 24 h), and then stimulated neuronal cells with Erastin (5 uM, 12 h) to explore whether inhibition of NRF2 pathway could weaken trehalose-ferroptosis suppression effect. Western blot results showed that ML385 significantly down-regulated the high expression of NRF2 protein induced by trehalose, and also inhibited the protein expression of the downstream target gene HO-1 of NRF2 (Figure 7F, 7G). The immunofluorescence staining of NRF2 also further confirmed that ML385 inhibited the high expression of NRF2 protein induced by trehalose (Figure 7H, 7I). In addition, ML385 can inhibit the increase in Gpx4 expression and the decrease in Acsl4 expression induced by trehalose (Figure 7F, 7G). At the same time, ML385 can significantly reduce the high-level GSH content induced by trehalose and increase the intracellular Fe^2+^ content and MDA level (Figure 7J–7L). The results from CCK8 and PI staining also confirmed that ML385 reversed the increase in cell viability promoted by trehalose (Figure 7M–7O). In summary, these results indicate that the NRF2/HO-1 pathway is involved in the inhibitory effect of trehalose on ferroptosis.

## DISCUSSION

Spinal cord injury (SCI) leads to irreversible tissue loss and neurological dysfunction [28, 29]. After SCI, neurons, glial cells and blood vessels are immediately mechanically damaged. Within hours to days after SCI, a series of complex secondary injury events begin to occur, including blood-spinal cord barrier permeability, ion imbalance, edema, glutamate excitotoxicity, lipid peroxidation, and inflammation [30]. In this study, we showed for the first time that trehalose plays a key protective role in acute SCI by inhibiting ferroptosis and ferroptosis-related inflammation. More specifically, we found that trehalose inhibited the expansion of cavities in the nerve tissue of mice with SCI, inhibited neuron loss, and improved functional recovery. In terms of mechanism, our results indicate that the neuroprotective effect of trehalose is due to the activation of the NRF2/HO-1 pathway, which in turn inhibits ferroptosis and ferroptosis-related inflammation. Our findings provide important insights into the previously unknown role of trehalose in SCI.

Trehalose is a natural disaccharide found in fungi, invertebrates and plants. It accumulates in large quantities under various pressures (including high temperature, low temperature, dryness, dehydration and oxidation) from these species to protect the integrity of cells from damage [31]. In addition, its non-toxic properties make it a good pharmacological agent. In addition to the attractive autophagy activation effect, there is evidence that trehalose can be used as a chemical chaperone to stabilize protein structure through direct interaction with proteins and to reduce protein misfolding and aggregation [32, 33]. Trehalose may also inhibit inflammation by regulating the NF-κB pathway [34]. Studies have found that trehalose can induce autophagy and act as a natural antioxidant, which can improve mitochondrial dysfunction and endoplasmic reticulum stress [23, 24]. In addition, after having been approved by the US Food and Drug Administration, trehalose has been regarded as a safe food ingredient by the European regulatory system [35]. A study showed that the combined application of cABC and trehalose to traumatic spinal cord injury can improve functional recovery [36]. Recently, Zhou et al. found that trehalose can also enhance the recovery of SCI through mTOR-independent activation of autophagy [22]. Our results are consistent with previous studies. Trehalose treatment can reduce the size of the damaged area in mice with SCI and promote tissue preservation and neuronal cell survival after SCI. Unlike the previous studies, we used a variety of different functional behavior experiments (BMS score, inclined plate test, rotating rod test, footprint test, and swimming test) to study the neuroprotective effects of trehalose. The experimental results more accurately verified the effect of trehalose on the recovery of motor function in mice with SCI. The multiple functional behavior experiments results show that the mice receiving trehalose treatment have better recovery of motor function, showing obvious gait recovery and improvement of motor coordination ability.

Ferroptosis is a cell death pattern, newly discovered in recent years. Ferroptosis is an oxidizing cell death induced by small molecules, is an iron-dependent process and is caused by the imbalance between the generation and degradation of lipid ROS in cells [37]. Ferroptosis inducers directly or indirectly act on glutathione peroxidase through different pathways, resulting in decreased cellular antioxidant capacity, accumulation of ROS, and finally oxidative cellular death [6, 7]. Studies have reported that ferroptosis is related to various tumors, ischemia-reperfusion injury, neurodegenerative diseases and other diseases. Activation or inhibition in the occurrence of ferroptosis can interference with the occurrence and development of diseases [38, 39]. Previous studies have proven that inhibiting ferroptosis can promote functional recovery after SCI [12, 13, 15]. In this study, we also found that the necrotic neurons and the number of iron-positive cells increased significantly after SCI, in and around the area respectively. Trehalose treatment can significantly inhibit the number of iron-positive cells and necrotic neuronal cells, indicating that trehalose may exert a neuroprotective effect by inhibiting ferroptosis of the neuronal cells. The series of experiments performed *in vivo* and *in vitro* indeed confirmed our conjecture that trehalose can inhibit the massive production of ROS and lipid peroxides after SCI. The Western blot and PCR results of ferroptosis-related proteins (Acsl4 and Gpx4) further confirmed our hypothesis. Trehalose can inhibit the production of ROS and ferroptosis caused by lipid peroxides after SCI, reduce neuronal cell degeneration and iron accumulation, and promote the recovery of motor function in mice after SCI.

Neuroinflammation is a significant response of SCI to trauma [40]. The first cells involved in the inflammatory response are microglia and neutrophils. They may cause tissue damage by releasing pro-inflammatory mediators such as IL-1β, IL-6 and TNF-α [41, 42]. The process of ferroptosis is often accompanied by inflammatory reactions. In the kidneys of mouse models with oxalate-induced acute kidney injury, inflammatory reactions related to ferroptosis were found, indicating that ferroptosis is closely related to inflammatory reactions [27]. Inflammatory mediators produced by lipid peroxidation and arachidonic acid metabolism are present in iron dead tissues [43]. Studies have shown that ferroptosis is related to the increased expression and release of prostaglandin-endoperoxide synthase 2 (PTGS2). Ferroptosis can directly increase the expression of PTGS2, accelerate the metabolism of arachidonic acid, and promote the secretion of inflammatory signaling molecules [44]. Therefore, we further studied the effect of trehalose on neuroinflammation after SCI. Our research shows that after trehalose treatment, the levels of IL-1β, IL-6 and TNF-α in CSF and spinal cord tissue are reduced. In addition, the immunofluorescence staining of the microglia marker CD11b also showed that trehalose can inhibit the accumulation of CD11b-positive microglia in the spinal cord injury area. Our research preliminarily proves that trehalose can inhibit the neuroinflammatory response after SCI, but whether it is an inflammatory response related to ferroptosis requires further in-depth research.

The NRF2 protein is considered to be the main regulator of cellular defense mechanisms against injury [45, 46]. Its function is to endow cells with a protective effect against oxidative stress, and it plays an important role in the transcriptional activation of a series of antioxidant and detoxification genes [47]. During oxidative stress, the interaction between NRF2 and Keap1 in the cytoplasm is disrupted [48]. NRF2 translocates to the nucleus and interacts with ARE, leading to the transcriptional induction of some cell defense genes, such as phase II detoxification enzymes HO-1, NQO1, and direct ROS scavenging proteins (GPx, SOD, CAT) [49, 50]. Many studies have confirmed that activation of NRF2 can prevent damage caused by ferroptosis [14, 24, 51]. Here, our data demonstrated that trehalose treatment activated the NRF2/HO-1 pathway in the occurrence of neuronal ferroptosis, and inhibition of the NRF2 pathway significantly reversed the inhibition of trehalose on ferroptosis, indicating that trehalose alleviated ferroptosis after SCI by inhibiting the NRF2 pathway.

In summary, we proved for the first time that trehalose can reduce the degeneration and iron accumulation of neuronal cells by inhibiting the production of ROS and ferroptosis caused by lipid peroxides after SCI, thereby promoting neuronal cell survival and improving the recovery of motor function. In terms of mechanism, our results indicate that the neuroprotective effect of trehalose is due to the activation of the NRF2/HO-1 pathway, which in turn inhibits ferroptosis and ferroptosis-related inflammation. Our findings provide important insights into the previously unknown role of trehalose in SCI, as well as new evidence supporting the hypothesis that suppression of ferroptosis plays a key neuroprotective role in SCI.
